# Lower limb strength training in children with cerebral palsy – a randomized controlled trial protocol for functional strength training based on progressive resistance exercise principles

**DOI:** 10.1186/1471-2431-8-41

**Published:** 2008-10-08

**Authors:** Vanessa A Scholtes, Annet J Dallmeijer, Eugene A Rameckers, Olaf Verschuren, Els Tempelaars, Maartje Hensen, Jules G Becher

**Affiliations:** 1 Department of Rehabilitation Medicine, VU University Medical Center, Amsterdam, the Netherlands; 2 EMGO Institute, VU University Medical Center, Amsterdam, the Netherlands; 3 Rehabilitation Foundation Limburg, Valkenburg, the Netherlands; 4 The Center of Excellence, Rehabilitation Center "De Hoogstraat", Utrecht, the Netherlands; 5 Rehabilitation Centre Heliomare, Wijk aan Zee, the Netherlands; 6 The Mytyl and Tyltylschool and Rehabilitation Center Amsterdam, the Netherlands

## Abstract

**Background:**

Until recently, strength training in children with cerebral palsy (CP) was considered to be inappropriate, because it could lead to increased spasticity or abnormal movement patterns. However, the results of recent studies suggest that progressive strength training can lead to increased strength and improved function, but low methodological quality and incomplete reporting on the training protocols hampers adequate interpretation of the results. This paper describes the design and training protocol of a randomized controlled trial to assess the effects of a school-based progressive functional strength training program for children with CP.

**Methods/Results:**

Fifty-one children with Gross Motor Function Classification Systems levels I to III, aged of 6 to 13 years, were recruited. Using stratified randomization, each child was assigned to an intervention group (strength training) or a control group (usual care). The strength training was given in groups of 4–5 children, 3 times a week, for a period of 12 weeks. Each training session focussed on four exercises out of a 5-exercise circuit. The training load was gradually increased based on the child's maximum level of strength, as determined by the 8 Repetition Maximum (8 RM). To evaluate the effectiveness of the training, all children were evaluated before, during, directly after, and 6 weeks after the intervention period. Primary outcomes in this study were gross motor function (measured with the Gross Motor Function Measure and functional muscle strength tests) and walking ability (measured with the 10-meter, the 1-minute and the timed stair test). Secondary outcomes were lower limb muscle strength (measured with a 6 RM test, isometric strength tests, and a sprint capacity test), mobility (measured with a mobility questionnaire), and sport activities (measured with the Children's Assessment of Participation and Enjoyment). Spasticity and range of motion were assessed to evaluate any adverse events.

**Conclusion:**

Randomized clinical trials are considered to present the highest level of evidence. Nevertheless, it is of utmost importance to report on the design, the applied evaluation methods, and all elements of the intervention, to ensure adequate interpretation of the results and to facilitate implementation of the intervention in clinical practice if the results are positive.

**Trial Registration:**

Trial Register NTR1403

## Background

Cerebral palsy (CP) is the most common cause of movement disability in childhood, with an incidence of 1.5–2.5 per 1000 live born children[1]. It is a non-progressive disorder that covers a number of neurological conditions, resulting in an abnormal development of movement and postural control[2]. From the perspective of the International Classification of Functioning, Disability and Health (ICF)[3], CP patients present with impairments in body function such as spasticity, low muscle strength, and selective motor control. These impairments may limit the performance of activities and participation in daily life. Improving and optimizing activities and participation are important treatment goals for therapeutic interventions.

A recent review has shown that low muscle strength, and not spasticity, causes the greatest limitations in motor function in children with CP[4], and this has shifted the focus from spasticity management towards strength training for these children. To be successful, strength training must be individualized, and should involve a progressive increase in intensity, thereby stimulating strength gains that are greater than those associated with normal growth and development (i.e. "overload")[5]. This is known as Progressive Resistance Exercise (PRE)[6], and for this type of exercise any method can be used to bear, overcome or resist force, such as body weight, free weights or machines.

Until recently, PRE was thought to be inappropriate, or potentially dangerous for children with CP because of the unfounded assumptions that such training would increase spasticity[7]. However, this concern is not supported by the results of recent studies which have shown that PRE strength training programs can improve lower limb muscle strength in patients with CP without increasing spasticity [8-11]. These results have been summarised in recent reviews [12-14], in which it was further concluded that PRE strength training *can *increase muscle strength, but that the effects are probably over-estimated because of the low methodological quality of these studies[12], and that future studies should furthermore develop more functional training programs aiming at a maximal carry-over into everyday activities[13].

Recently, new randomized clinical trials (RCT's) have been carried out to evaluate the effect of this type of functional strength training[10,15,16] in children with CP. Conflicting results were found on isometric muscle strength[15,16], gross motor function[10,15,16] and walking ability[10,16]. This might be due to the slightly different evaluation methods, but more probably to the differences in relevant training characteristics, such as type of training (e.g. home or school based), intensity (e.g. load based on body mass or repetition maximum), progression (e.g. none, weekly, individually based) and duration of the training (e.g. 5 or 12 weeks). Unfortunately, this information was not always provided, which hampers correct interpretations. For an adequate interpretation of the effectiveness of the intervention, standardisation and reporting on all relevant aspects of the training is therefore of utmost importance.

For PRE strength training, the key principle should always be standardized: the timely progression in strength intensity based on the child's *individual *level of strength, to ensure the principle of progressive overload[17,18]. This is best assessed by the repetition maximum (RM), which is the maximum number of repetitions that can be performed correctly under a given load. The heaviest load with which an exercise can be performed for 1 complete repetition with correct performance is the 1 RM. According to current guidelines, training should start with a dynamic warm-up period, initially 1 or 2 sets of 8–15 repetitions with a light to moderate load (about 30–60% 1 RM) to learn the right technique, and then progress to 3–5 sets of 8–15 repetitions[19]. Loads can safely be progressed to 70–85% of 1 RM[20]. A training frequency of at least 2 non-consecutive days per week is further recommended. In addition, for children the training should be fun, and group-training is thought to increase both the fun and the individual motivation to progress.

It is therefore thought that individualized, but group-given, school-based, functional PRE strength training, with sufficient frequency, intensity and progression, increases the effectiveness of the training, although these aspects need further investigation. The purpose of this study was to evaluate, in an RCT, the effectiveness of such functional PRE lower limb strengthening program in a group of children with CP. A protocol was therefore developed to train lower limb muscle strength, based on the current guidelines for PRE strength training in healthy adults[6] and children[17,19], the recent literature on strength training for CP [12-14], and the expertise of experienced paediatric physical therapists in the Netherlands. In this protocol, the frequency, duration, weekly intensity and progression on the basis of RM testing of the training, and also the type and technique of each of the exercises were standardized. We hypothesize that children who will follow this structured functional PRE strength training program will increase in muscle strength, which accordingly will lead to functional improvement in gross motor function and walking ability, but with no negative effect increasing spasticity or decreasing range of motion, compared to children receiving usual care. This paper describes the study design and all relevant elements of the functional PRE strength training protocol.

## Methods

### Design

The study has a single-blinded, randomized controlled design and the study protocol was approved by the Medical Ethics Committee of the VU University Medical Center in Amsterdam, the Netherlands.

### Setting

The training and assessments took place in three special schools for physically disabled children in the Netherlands, from which all the children were recruited.

### Participants

All the participants were ambulatory children with spastic unilateral or bilateral CP[2,21]. The inclusion criteria were: 1) age between 6 and 13 years; 2) able to accept and follow verbal instructions; 3) ability to walk independently indoors, with or without walking aids (Gross Motor Function Classification System [GMFCS][22] levels I – III); 4) able to participate in a group training program. Children were excluded if they had instable seizures, if they had received treatment for spasticity or surgical procedures up to 3 months (for botulinum toxin type A injections) to 6 months (for surgery) prior to the study (or planned in the study period); if any change in medication was expected during the study period; or if they suffered from other diseases that interfered with physical activity.

A paediatric physiatrist working in each school pre-selected the children, based on the inclusion and exclusion criteria. The parents of the children who met these criteria received written information about the study. Accordingly, the parents of each potential participant were contacted by a researcher who gave further oral explanation, and answered any questions, if necessary. Written informed consent was given by the parents, and all children who were over 12 years of age. After all the informed consents had been collected, randomization was performed per school. The children were randomly allocated according to three stratification variables: GMFCS level (I; II–III); age (youngest: 6–9 years; oldest 10–13 years), and gender (boy; girl). Families were notified by means of a letter of their child's allocation to the intervention group or the control group prior to the first baseline assessment.

### Sample size

At least 42 children were required to detect a difference in improvement of 5% on the Gross Motor Function Measure (GMFM) between the intervention group and the control group, with a power of 0.8 and an alpha of 0.05. To allow for some drop-outs, we aimed to recruit at least 50 children (25 intervention, 25 control).

### Intervention: Lower Limb Strength Training

The intervention group followed a 12-week functional PRE strength training program (not including holidays), 3 times a week. Each training session lasted for 45–60 minutes.

The training replaced the conventional physical therapy program, when this was consistent with the objectives of the strength training (improvement of functional abilities by increasing muscle strength/fitness), but was additional to any other therapies (e.g. swimming, occupational therapy). The strength training was specifically designed to strengthen the anti-gravity muscles of the lower limbs (such as the glutei, vasti, gastrocnemius, soleus), while performing functional exercises in a circuit training.

#### Circuit training

The training sessions were held in small groups (± 4–5 children) with a 5-station circuit, supervised by two experienced physical therapists per group. During each training session, the children wore their regular (street) shoes, splints, trousers, and they all wore a group T-shirt to stimulate the group-feeling. Each training session started and finished with a warming up and cooling down period of 5–10 minutes, during which muscle stretching exercises and aerobics were performed. During the main training phase, each child performed 4 different exercises on the 5-station circuit. The different stations of the circuit were named as followed: leg-press, loaded sit-to-stand, loaded game, unloaded game and relax. Table 1 presents the characteristics of the 5-station circuit.

**Table 1:** Characteristics and training volumes of the 5-station circuit

**Station**	**Load**	**Trained Leg**	**Exercise**	**Functional**	**Supervision**	**Game**	**Resistance**
1. Leg-press	High	Bilateral	Leg-press	No	Very strict	No	Leg-press
2. Loaded sit-to-stand	High	Bilateral	Sit-to-stand	Yes	Very strict	No	Weight vest
3. Loaded game	Low	Unilateral	Half-knee rise; Lateral step up; Forward step up; (Sit-to-stand)	Yes	Strict	Yes	Weight vest
4. Unloaded game	No	Unilateral	Half-knee rise; Lateral step up; Forward step up; (Sit-to-stand)	Yes	Strict	Yes	Body weight
5. Relax	n.a.	n.a.	n.a.	n.a.	none	n.a.	n.a.

Abbreviation: n.a. = not applicable

The exercise stations were a combination of high load (e.g. leg-press and loaded sit-to-stand stations), low load (e.g. loaded game station) and no load (e.g. unloaded game station) exercises (see Table 1; the exercises are explained in more detail in Additional files 1 to 4).

At the stations that were highly loaded (e.g. leg-press and loaded sit-to-stand stations), both legs were trained simultaneously by means of bilateral exercises. The exercises at these stations were fixed: a leg-press exercise (see Additional file 1) and a loaded sit-to-stand exercise (see Additional file 2). Of these, only the loaded sit-to-stand is a functional exercise, resembling an everyday activity with which ambulatory children with CP may experience difficulty (i.e. rising from a chair).

At the stations that were low or not loaded (e.g. loaded game station and unloaded game station), the most affected leg was (preferably and if possible) trained by means of unilateral exercises. All exercises at these stations were functional. These exercises were randomly chosen by the physical therapist: a lateral step up exercise or a forward step up exercise (i.e. climbing a stair or stepping up onto a kerb, see Additional file 3), or a half-knee rise exercise (i.e. rising from the ground, see Additional file 4). If a child was not able to perform these unilateral exercises, the sit-to-stand exercise (see Additional file 2) could be performed instead.

At the stations that were highly loaded (e.g. leg-press and loaded sit-to-stand stations), correct performance of the exercises was mandatory and required very strict supervision (close one-on-one guidance per child by the physical therapist). At the stations that were less or not loaded (e.g. loaded game station and unloaded game station), somewhat less supervision was needed for these exercises (one-on-two guidance per two children by the physical therapist, so that the children could perform the exercises together). Furthermore, it was also important that the children enjoyed the training. Therefore, the exercises at the loaded game and unloaded game stations could be chosen at random. In addition, the exercises at these stations were suitable to be integrated in a game-like situation (for example: playing skittles), which could be performed together or as a competition.

**Figure 1:**
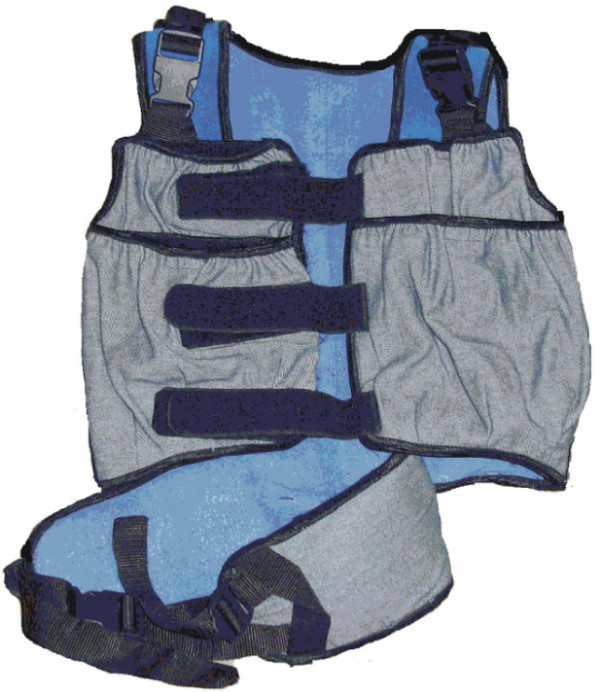
Weight vest.

At the leg-press station resistance was applied by the leg-press machine itself, which was specifically adapted for children by means of an elevated footplate (EN-Dynamic Seated Leg Press, Enraf Nonius, The Netherlands). At the other loaded stations, where functional exercises were performed, resistance was applied by means of a customised weight vest (see Figure 1).

#### Training volume

Except for the relax station, which served as a relax moment, each station had its own training volume (i.e. a combination of load, repetitions, sets and rest) with which the exercise was performed.

**Figure 2:**
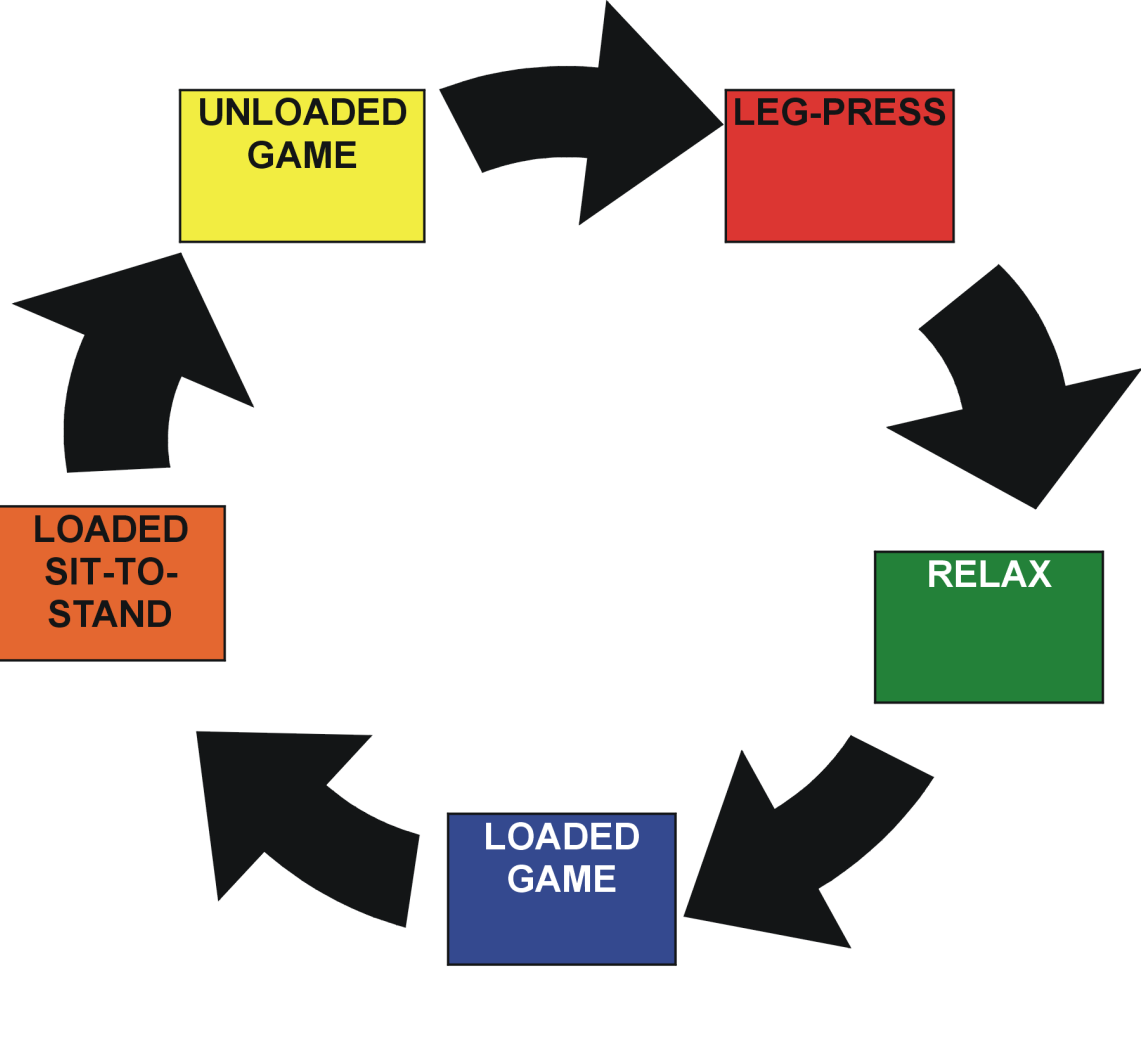
**The 5-station circuit**. During one training session, in which a maximum of 5 children participated, each child started at one of the 5 stations, and rotated through the circuit until 4 exercises had been completed.

As is shown in Table 2, the choice of a training volume is directly related to the desired goal of muscle training, and to achieve enhanced muscle strength, the training must be based on a fixed combination of a high load (70–95% 1 RM), 8–12 repetitions, 1–3 sets and sufficient rest to allow for muscle recovery. In this study we focussed on the improvement of both strength and strength/endurance training, and therefore used a high to low load and a low to medium number of repetitions. Table 3 presents the training volumes of the exercises at the different stations. All exercises were performed in 3 sets of 8 repetitions, with a 90 second rest in between the sets. Each exercise was performed within 7–10 minutes. At the beginning of each new training session, every child started at one of the 5 stations, from which he/she rotated to the next, until he/she had completed 4 stations (see Figure 2). At the beginning of each subsequent training session, the child started at a different station. Consequently, each station was completed, i.e. each exercise was performed, 2 to 3 times a week.

**Table 2:** Different training volumes, related to the desired goal of muscle training (after Wilmore & Costill Physiology of Sport and Exercise)

**Training goal**	**Training volume **			
	***Load (% 1 RM)***	***Repetitions***	***Sets***	***Rest between sets***
Maximum strength training	95–100%	1–3	1–3	2–4 min
(Sub-maximal) strength training	70–95%	8–12	1–3	90–120 sec
Strength endurance training	50–70%	10–15	1–3	45–90 sec
Endurance training	<50%	20–50	3–5	<45 sec
Co-ordination training	<30%	30–70	4–6	<45 sec

Abbreviation: RM = Repetition Maximum

**Table 3:** Training volumes of progressive resistance exercise

**Station**	**Training volume **			
	***Maximum Load***	***Repetitions***	***Sets***	***Rest***
1. Leg-press	100% 8 RM	8	3	90 sec
2. Loaded sit-to-stand	75% 8 RM	8	3	90 sec
3. Loaded game	25% 8 RM	8	3	90 sec
4. Unloaded game	body weight	8	3	90 sec
5. Relax	n.a.	n.a.	n.a.	n.a.

Abbreviation: RM = Repetition Maximum, n.a. = not applicable

After each training session the physical therapist recorded the type of exercise performed, with the number of repetitions and sets, and the training load in a child-specific diary. Any adaptations made to the training were also recorded, as well as any muscle soreness or any other lower limb pain that the child had possibly experienced.

#### Training load and 8 Repetition Maximum

The training load for the exercises was established according to the individual 8 RM test. The 8 RM is approximately equal to 80% 1 RM[23] (N.B.: the 1 RM test was considered to be too heavy). The 8 RM test procedures were initiated after the children had become familiar with the training program, and when they could perform the exercises correctly. To determine the 8 RM, the child started with 3 repetitions (no load) to practise the correct performance: each repetition had to be performed through full (possible) range of motion, with adequate speed (speed of repetition: extension movement: 2–3 seconds/flexion movement: 2–3 seconds[6]). Then, two warm-up trials of 3 repetitions were performed at 50% and 70% of the predicted 8 RM, respectively. The predicted 8 RM is based on the child's body weight, according to GMFCS-specific guidelines which we established in an earlier pilot study (see Table 4). After the third and actual test trial, the child was instructed to perform the trial at 100% of the predicted 8 RM until (temporary) muscular exhaustion, or until a maximum of 10 repetitions. If a repetition was performed incorrectly, it was not counted; and if two consecutive repetitions were performed incorrectly, the trial was ended. The criteria for incorrect technique were: without full (possible) range of motion, with incorrect technique and inadequate speed (i.e. too slow/fast). If a child performed less than 6 or more than 10 correct repetitions, a 5 to 10% load was either reduced or added, respectively. After a 3 minute rest, the trial was repeated until the child was able to perform 7–9 repetitions, but no more.

**Table 4:** Guidelines for the predicted 8 repetition maximum

**Station**	**GMFCS level**	**Predicted 8 RM**
1. Leg-press	I	120% of the body weight
	II	100% of the body weight
	III	80% of the body weight
2. Loaded sit-to-stand	I	35% of the body weight
	II	30% of the body weight
	III	25% of the body weight

Abbreviations: GMFCS = Gross Motor Function Classification System; RM = Repetition Maximum

RM test procedures are known to be labour-intensive and time-consuming, and to require close and experienced supervision[19]. Therefore, we could not perform these tests for every exercise on a regular basis. We chose to regularly determine the 8 RM for the leg-press[24] (i.e. weeks 3, 6, 8 and 10) (see Additional file 5), but the 8 RM for the loaded sit-to-stand[25] was only tested twice (i.e. weeks 4 and 9). In two other weeks (7 and 11) the training load for the loaded sit-to-stand was estimated based on the progress in 8 RM testing on the leg-press between weeks 3 and 6 and weeks 8 and 10, respectively (see Additional File 5), which was set at a minimum of 0% and a maximum of 10%. No 8 RM test was determined for the loaded game exercises. The load for these exercises was estimated with the 8 RM test for the loaded sit-to-stand (see Additional File 5).

Two types of loading or resistance materials were used: the leg-press with a weight range from 5 to 200 kg, adjustable in 1 kg steps, and a weight vest (see Figure 1) with weight bags in different weights, ranging from 0.5 kg – 3 kg. The load in the weight vest was always equally divided between the front and the back and between the left and right side.

Each exercise had its own maximum training load (see also Table 3): the leg-press and loaded sit-to-stand exercises were performed at a maximum of 100% 8 RM and 75% 8 RM, respectively, the loaded game at 25% 8 RM, and the unloaded game with no extra resistance except body weight. These training loads were adjusted to new individual levels of strength, if necessary (i.e. as determined by the 8 RM test). The first 6 weeks were intended to slowly build up the training towards these maximum training loads and during the last 6 weeks the children were training at the maximum load. The exact training loads and timing of 8 RM tests per exercise per week are presented in Additional File 5.

### Outcome measures

The data were collected in each of the participating schools by two blinded, independent research assistants. One pre- (T0) and three post-training assessments were made (T 1/2: after 6 weeks of training, T1: at the end of the training, T2: 6 weeks after the end of the training). The T0, T1 and T2 assessments were scheduled during a two-week period and the T 1/2 assessment was scheduled during a one week period. Measurements of the height and weight of the children were obtained during each session, as well as the type of shoes they wore and the type of orthosis or walking aid they used.

**Figure 3:**
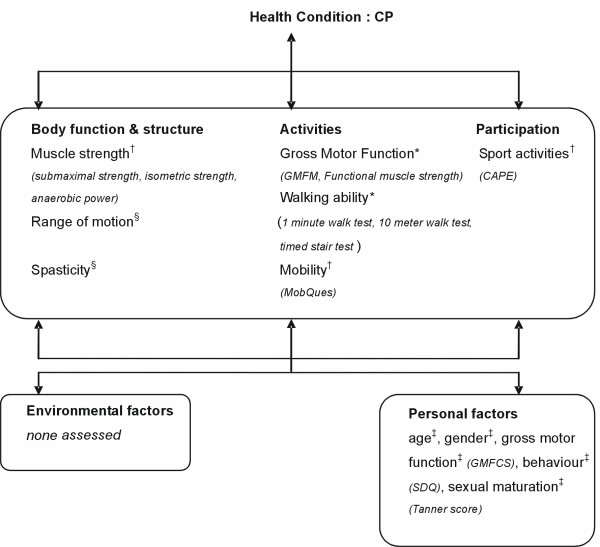
**Study outcomes and instruments used on the different ICF levels**. * primary outcomes; ^† ^secondary outcomes; ^§ ^adverse outcomes; ^‡ ^control outcomes. Abbreviations: CP = Cerebral Palsy, GMFM = Gross Motor Function Measure, CAPE = Children's Assessment of Participation and Enjoyment, MobQues = Mobility Questionnaire, GMFCS = Gross Motor Function Classification System, SDQ = Strength and Difficulties Questionnaire

The outcome measurements in this study include assessments of the body function and structure, and activity and participation at ICF level (see Figure 3). The primary outcomes were gross motor function and walking ability (activity level), and the secondary outcomes were muscle strength (body function and structure level), mobility (activity level) and sport activities (participation level). In addition, various personal characteristics were measured once at baseline, in order to control for possible age, gender and gross motor function differences, as well as for emotional or behavioural problems (measured with the Strength and Difficulties Questionnaire [SDQ][26]) and degree of sexual maturation (based on the Tanner score[27]).

### Primary outcome measures

#### Gross Motor Function

*The Gross Motor Function Measure (GMFM) *is a standardized observational measurement instrument that requires a child to demonstrate various motor skills, as outlined in the GMFM administration and scoring guidelines. Consequently, it reports on the child's actual level of ability. It has been validated for evaluating change in the gross motor activities of children with CP. In this study we used the 66-item version of the GMFM (GMFM-66), an internationally recognised valid and reliable outcome measure, based on interval scaling[28].

*Functional muscle strength *was measured with two different functional exercises in which the large muscle groups that are important in daily activities were assessed: the 30-s Lateral Step-Up Test[29] and the 30-s Sit-to-stand Test[29].

The 30-s Lateral Step-Up Test[29] assesses the number of step ups that the child can perform in 30 seconds. The test was performed on a 21 cm (GMFCS I and II) or an 11 cm (GMFCS III) step. The child stood next to the step and put the more impaired (i.e. tested) leg on the step, where it remained throughout the entire test. The child was asked to lift his/her less or un-impaired (i.e. non-tested) leg up and put it on the step, by fully extending the hip and knee of the tested leg. After this, he/she set the non-tested leg back on the ground, next to the step. A full movement (stepping up and down) was counted as one repetition.

The 30-s Sit-to-Stand Test[29] assesses the number of sit-to-stands that the child can perform in 30 seconds. The test was performed on a child-sized chair with a height-adaptable seat (no backrest, no armrest). The upper legs were parallel to the floor, the feet parallel on the ground (as flat as possible) and the trunk erect. The child was asked to stand up, as erect as possible with symmetric hip strategy ("flex hips and move trunk forward until the shoulders are above the knee joint and then stand up"). A full movement (standing up and sitting down) was counted as one correct repetition.

#### Walking ability

*The Timed 10-Meter Walk test (10 MWT) *assessed the time (in seconds) and number of footsteps needed to walk 10 meters. The 10 MWT test was performed on a 14 meter straight, flat, smooth, non-slippery walking surface. The child was instructed to walk at a self-selected comfortable speed. The test was performed with a 'flying start' (i.e., while the child walked approximately 14 meters, the time and number of footsteps for walking the intermediate 10 meters were measured). From this, cadence, step-length and comfortable walking velocity were calculated.

*The 1-Minute Walk Test*[30]*(1 MWT) *assesses the distance (in meters) walked during 1 minute. The 1 MWT test was performed on a 20–22 m figure of eight-shaped, flat, smooth, non-slippery oval walking track. The child was instructed to walk around the track for 1 minute at his/her fastest attainable speed (no running). During this test the distance (in meters) was calculated to the nearest meter by means of self-attached meter marks on the track, to calculate fast walking speed.

*The Timed Stair Test (TST) *assesses the time needed to go up and down stairs[15]. The test was performed on a 4 or 5-step set of stairs, with handrails on both sides. The child started at the bottom of the stairs, and was instructed to walk up the stairs as fast as possible, without running, and with alternating feet if possible (if not: step with both feet on the same step), then turn round on the platform and walk down the stairs, with alternating feet if possible (if not: step with both feet on the same step).

### Secondary outcome measures

#### Muscle strength

#### 

*6 RM muscle strength.* The strength of the major muscles of the lower body, as a percentage of body weight, was measured with a 6 RM test on a leg-press. This test was chosen as an outcome measure instead of the 8 RM test, as a slight modification of the training exercise (N.B.: the 1 RM test was considered to be too heavy). The 6 RM test was performed in the same way as the 8 RM test described in the Methods section, except that the child was instructed to perform the third trial at 100% of the predicted 6 RM until temporary muscular exhaustion, or until a maximum of 8 repetitions (range 5–7). Based on an earlier pilot study, the initial leg-press load for the 6 RM was estimated at 130%, 110% and 90% of body weight for children with levels GMFCS I, II and III, respectively.

#### 

*Isometric strength tests*. The isometric muscle strength of the unilateral hip flexor/abductor, knee flexor/extensor, and ankle plantarflexor muscles were measured with a hand-held dynamometer (MicroFet, Biometrics, Almere)[15,31]. A "make" test was performed, in which the investigator stabilized the dynamometer, while the child pushed as hard as possible against the dynamometer for a period of 3 seconds, during which the peak force (in Newton) was assessed. The mean scores of three tests were used for the analysis. A standardized protocol was used for positioning of the child, joint fixation, joint positioning and dynamometer resistance (see Table 5).

**Table 5:** Isometric muscle strength testing: protocol for child positioning, joint fixation, joint positioning and dynamometer resistance

**Muscle group**	**Child position**	**Joint fixation**	**Joint starting position**	**Position of dynamometer resistance**
Knee extensors	Sitting	Pelvis and thigh	Knee flexed 90°	Anterior tibia, 5 cm proximal to malleoli
Knee flexors	Sitting	Pelvis and thigh	Knee flexed 90°	Posterior calf, 5 cm proximal to malleoli
Hip flexors	Sitting	Pelvis	Hip flexed off surface	Anterior thigh, 3 cm proximal to patella
Hip abductors	Supine	Pelvis	Hip slightly flexed off surface using a knee roll	Lateral thigh, 5 cm proximal to the knee joint
Ankle plantarflexors	Supine	Pelvis and lower leg	Hip flexed 90°, lower leg stabilized on a bench, ankle in neutral position.	Plantar surface of foot, across metatarsal heads

Abbreviation: cm = centimeter

#### 

*Anaerobic power*. The anaerobic sprint power output of the legs was evaluated during a 20-second full out cycle-test (adapted from the original 30-second test)[32]. It was performed on a child-adapted cycle-ergometer (Corival, Lode bv, Groningen). After a warming-up period of 5 minutes (cycling at low resistance, interspersed with three 5-second sprints), the child was asked to cycle as fast as possible for 20 seconds (sprint) against a constant force. Two performance indices were calculated (mean and peak power) with specialized software (ProCare bv, Groningen). The former reflects the child's ability to sustain high power, whereas the latter indicates the ability to produce high mechanical power in a short time. These outcomes are considered to be closely related to the short-term energy system that is employed to perform daily tasks in children with CP[32].

#### Mobility

*The Mobility Questionnaire (MobQues) *is a Dutch questionnaire that measures mobility limitations based on 47 caregiver-reported items. These concern the mobility tasks of every day life, which include both indoor and outdoor activities such as 'sit down on a chair', 'go up stairs', and 'walk on asphalt'. One of the child's parents or caregivers was asked to complete the MobQues, and to indicate how difficult it was for their child to perform these mobility tasks in the usual way (with the use of assistive devices if needed) without any help from others. The response options, given on a 5-point scale, were: not difficult at all, slightly difficult, somewhat difficult, very difficult, impossible without help. In this study we used the 28-item version of (MobQues-28), which is based on interval scaling. The MobQues-47 and MobQues-28 were found valid and reliable according to a recent study that was performed in a Dutch population, which results' will be published in the near future.

#### Sport activities

*The Children's Assessment of Participation and Enjoyment (CAPE)*[33] measures the child's participation in every day activities, outside of the school program. It is completed by the parents or caregivers, together with the child. For the present study, 17 items were chosen from the sub-scales of physical (items 32, 36), recreational (items 16, 20, 21, 33–35, 37–41) and skill-based (items 17–19) activities as outcome measures, because these items measure the frequency of participation in 17 different sport activities. For each of these activities the child was asked whether, and if so, how often he/she had participated in this activity in the previous 4 months.

### Adverse outcomes

*The Range f Motion *in the hamstrings, adductors, rectus femoris, soleus and gastrocnemius muscles was assessed with goniometry during the third of three slow passive stretches (>3 seconds)[34].

#### 

*Spasticity*. Goniometry was also used to measure spasticity in the hamstrings, adductors, rectus femoris, soleus and gastrocnemius muscles by assessing the joint angle at which a 'catch' (defined as a sudden increase in muscle tone, blocking further movement) occurred in a fast passive stretch (<1 second)[35].

### Data-analyses

Student T-tests (continuous data, if normally distributed), Mann-Whitney tests (continuous data, if not normally distributed), and Chi-square tests (dichotomous and ordinal data) will be used to evaluate group differences at baseline. Differences in change from baseline between the intervention group and control group for the three post baseline assessments will be evaluated by means of generalized estimating equations (GEE) for longitudinal analysis[36]. GEE is appropriate for analyzing longitudinal data with hierarchically structured data and the advantage of GEE is that this method takes into account the dependency of repeated measures within the same person. To determine the effect of the strength training program, changes in the training group will be compared to the changes in the control group (interaction between group * time).

## Discussion

Randomized clinical trials are considered to present the highest level of evidence. However, until now, only three randomized trials have evaluated the effects of a PRE functional strength training program in children with CP. These studies have reported positive training effects, although their results are still not truly convincing with regard to their corresponding outcome measures. This might be due not only to a difference in the evaluation methods that were applied but also to a difference in training characteristics. The majority of studies do not present a detailed report on their training program. Not only does this hamper correct interpretation of the results, but it also hampers implementation of the intervention in clinical practise in clinical practice. So, for a correct interpretation of the effectiveness of an intervention, standardization and reporting on all relevant aspects of the training protocol is of utmost importance.

For PRE strength training in particular, every study should give a detailed description of the key principle of the PRE: the timely progression in strength intensity, based on the child's *individual *level of strength, to ensure the principle of progressive overload[17,18]. To guarantee overload based on the individual level of strength, it should preferably be assessed according to the Repetition Maximum (RM).

Furthermore, each intervention study should also standardize and describe other relevant aspects such as frequency, duration and weekly volume of the training, and also the type and technique of each of the exercises.

Nevertheless, children with CP may present with a variety of motor disorders (e.g. poor co-ordination, loss of selective motor control), orthopaedic problems and other associated conditions (e.g. disturbances of cognition)[2], which often makes individual adjustments in the performance of the exercises necessary. Despite this, individual standardization of the training *should *and *can *be applied, in order to meet the requirements of progressive overload.

This paper outlines the design and all relevant aspects of the training protocol of a randomized controlled trial examining the effects of a school and group-based functional strength training program for children with CP. Our study was based on the hypothesis that an individualized, but group and school-based, functional PRE training will strengthen the lower limbs, and will accordingly lead to functional improvements in gross motor function and walking ability in children with CP. The results of this trial will be presented as soon as they become available.

## Competing interests

The authors declare that they have no competing interests.

## Authors' contributions

VAS, AJD and JGB contributed equally to this work: they participated in the design, developed the training protocol, participated in the co-ordination, and drafted the manuscript.

EAR, OV, ET and MH also contributed equally to this work: they developed the training protocol, trained the participants, and participated in the co-ordination. All authors participated in the reviewing process and approved the final manuscript.

## Pre-publication history

The pre-publication history for this paper can be accessed here:



## Supplementary Material

Additional file 1 Leg-press exercise. This table describes the performance of the leg-press exercise Click here for file

Additional file 2 Sit-to-stand exercise. This table describes the performance of the sit-to-stand exercise. Click here for file

Additional file 3 Step-up exercises. This table describes the performance of the lateral and forward step-up exercises. Click here for file

Additional file 4 Half-knee exercise. This table describes the performance of the half-knee rise exercise. Click here for file

Additional file 5 Weekly training volumes and timing of the 8 RM test. This table describes the weekly training volumes and timing of the 8 RM test. Click here for file
