# Supplementary material for: Lower limb strength training in children with cerebral palsy – a randomized controlled trial protocol for functional strength training based on progressive resistance exercise principles
Source: BMC Pediatr. 2008 Oct 8;8:41. doi: 10.1186/1471-2431-8-41 (PMC2579291; doi:10.1186/1471-2431-8-41)
Supplement: Additional file 1 — Leg-press exercise. This table describes the performance of the leg-press exercise [file 1471-2431-8-41-S1.pdf]

**Leg-press**<br><br>Bilateral exercise	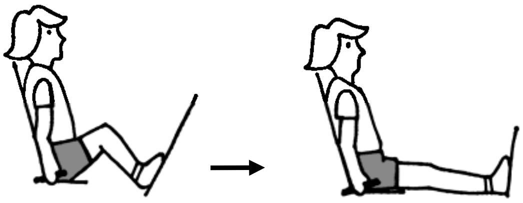
1 repetition:	1x extend & flex
Initial starting position:	Position: sitting with flexed hips and knees<br>Hands: holding the handgrips<br>Hips: flexion<br>Lower legs: knees bent to 90˚<br>Feet: evenly placed on the footpad, heels placed a little less than a shoulder width apart, making heel contact → also see *Adaptations*<br>Toes: pointing slightly outward
Instructions:	"Slowly push the footpad forward, keep knees slightly flexed, and bend back again slowly"
Trainer:	Trainer stands or sits beside the child.
Strategy:	Push the footpad forward by extending the legs (do not lock the knees!) and pushing the load with both feet equally. (The child may be helped by the trainer to initiate the very first movement)<br>Maintain the extended position for 1 second.<br>Slowly bend the legs back to the initial starting position to complete one repetition.<br>The back should remain flat against the machine's pad throughout the entire exercise.<br>Repeat 8 times.
Breathing	Exhale during the pushing action<br>Inhale during the return movement
Speed:	One extension per two to three seconds, and one flexion per two to three seconds. The trainer can count out loud (one-two-extend / one-two-flex) as a guidance.
Correct trial:	Extension of the legs while keeping the weight symmetrically on both legs and returning back to the flexed position without lifting the back from the machine's pad.
Incorrect trial:	Locking of the knees.<br>Extending the legs with an obviously asymmetrical posture.<br>Unable to maintain an extended position for 1 second.<br>Lifting the back from the machine's pad at some point in the exercise.<br>Returning to a flexed leg position abruptly without good control.
Adaptations<br>Initial starting position:	If the child has contractures or wears a rigid orthosis, heel contact is not possible. This cannot be adapted.
