# Supplementary material for: Lower limb strength training in children with cerebral palsy – a randomized controlled trial protocol for functional strength training based on progressive resistance exercise principles
Source: BMC Pediatr. 2008 Oct 8;8:41. doi: 10.1186/1471-2431-8-41 (PMC2579291; doi:10.1186/1471-2431-8-41)
Supplement: Additional file 2 — Sit-to-stand exercise. This table describes the performance of the sit-to-stand exercise. [file 1471-2431-8-41-S2.pdf]

**Sit-to-stand**<br><br>Bilateral exercise	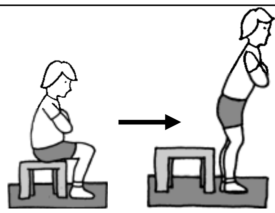<br>Sit-to-stand<br>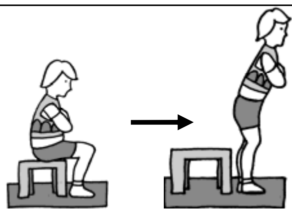<br>Loaded Sit-to-stand
1 repetition:	1x stand up & sit down
Initial starting position:	Position: sitting on chair<br>Hands: on waist or across the chest (with assistance: in hands of trainer).<br>Trunk: erect<br>Hips: 90˚ flexion (hips and knees levelled → thigh is parallel to the floor)<br>Knees: 105˚ flexion (full extension is defined as 0˚)<br>Feet: parallel, as flat on the floor as possible → also see *Adaptations*
Chair:	Type: no armrests, no backrest<br>Height: Adjusted to the initial starting position
Instructions:	“Stand up slowly. Stand still, then sit down again slowly. Do not use hands or support (if possible)”
Trainer:	Trainer stands beside or in front of the child. Support may be given for balance.
Strategy:	Move the trunk forward by flexion of the hips until the shoulders are above the knee joints.<br>Stand up.<br>Stay standing up for 1 second.<br>Sit down.<br>Repeat 8 times
Speed:	One stand-up per two to three seconds. One sit-down per two to three seconds.
Correct trial:	Standing up with as much symmetrical hip strategy as possible to the defined standing position, which requires the subject’s trunk and lower extremities to being fully extended
Incorrect trial:	Losing balance.<br>Standing up with an obviously asymmetrical posture during the test (i.e. head of child leans over one or both knees)<br>Unable to maintain a standing position for two seconds after standing up<br>swaying the trunk back and forth several times to initiate the task of standing up.<br>Sitting down abruptly without good control.
Adaptations<br>Initial starting position:	 Same as above, but decrease knee flexion to 100˚ - 120˚ (hips slightly higher than knees by raising the seat of the chair.
