# Supplementary material for: Lower limb strength training in children with cerebral palsy – a randomized controlled trial protocol for functional strength training based on progressive resistance exercise principles
Source: BMC Pediatr. 2008 Oct 8;8:41. doi: 10.1186/1471-2431-8-41 (PMC2579291; doi:10.1186/1471-2431-8-41)
Supplement: Additional file 3 — Step-up exercises. This table describes the performance of the lateral and forward step-up exercises. [file 1471-2431-8-41-S3.pdf]

**Lateral or forward step up**<br><br>Unilateral exercise<br>(Example: target left leg)	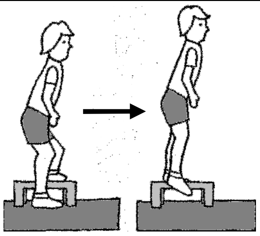 Lateral Step Up<br>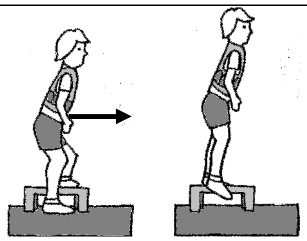 Loaded Lateral Step Up
1 repetition:	1 step up & 1 step down
Initial starting position:	Position: standing at about 10cm besides or in front of the step<br>Hands: on waist / across the chest or alongside body (with assistance: in hands of trainer)<br>Trunk: erect<br>Hips: neutral (as neutral as possible)<br>Left leg: flexed in hip and knee, foot placed on step<br>Right leg: hip and knee extended (as far as possible, full extension is defined as 0°), foot on floor, with heel contact, or as flat on the floor as possible → also see *Adaptations*
Step:	Height: GMFCS I & II : 40 - 20 cm<br>GMFCS III: 20 - 10 cm
Instructions:	"Step up slowly. Stand still, and step down again slowly. Do not use hands or support (if possible)"
Trainer:	Trainer stands in front of the child. Support may be given for balance.
Strategy:	Step up: place right foot up on the step, next to the left foot. Extend the knees as much as possible.<br>Pause: keep standing with both feet on the step for 1second.<br>Step down: place right foot down on the floor, leave left foot up on the step.<br>Repeat 8 times
Speed:	One step-up per one to two seconds. One step-down per one to two seconds.
Correct trial:	Stepping up and down with good balance, without tripping and without pulling up using the arms of the trainer.
Incorrect trial:	Losing balance.<br>Unable to maintain a standing position for one second on the step.<br>'Pulling' up using arms of trainer.
Adaptations<br>Initial starting position:	 If the child has contractures or wears a rigid orthosis, heel contact is not possible. This cannot be adapted.
