# Supplementary material for: Lower limb strength training in children with cerebral palsy – a randomized controlled trial protocol for functional strength training based on progressive resistance exercise principles
Source: BMC Pediatr. 2008 Oct 8;8:41. doi: 10.1186/1471-2431-8-41 (PMC2579291; doi:10.1186/1471-2431-8-41)
Supplement: Additional file 4 — Half-knee exercise. This table describes the performance of the half-knee rise exercise. [file 1471-2431-8-41-S4.pdf]

**Half Knee Rise**<br><br>Unilateral exercise<br>(Example: target right leg)	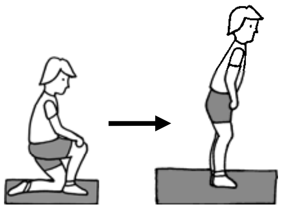 Half-knee rise 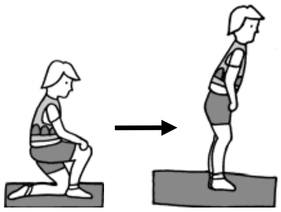 Loaded half-knee rise
1 repetition:	1 stand up & 1 return
Initial starting position:	Position: on floor (or mat) in half-kneeling position on left knee.<br>Hands: on waist, on knee (with assistance: in hands of trainer).<br>Trunk: erect or slightly forward.<br>Hips: Left: may range from 20˚ flexion to neutral (as long as buttocks are clear of lower legs / weight baring surface); Right: range from 70˚ - 110 ˚ flexion<br>Knees: Left: on floor in 90˚ - 130˚ flexion (full extension is defined as 0˚); Right: range from 70˚ - 90˚ flexion.<br>Feet: Left: on floor; Right: as flat on floor as possible, preferably heel contact → also see *Adaptations*
Instructions:	"Stand up slowly. Stand still, and slowly kneel down again on the left knee. Do not use hands or support (if possible)"
Trainer:	Trainer stands in front of the child. Support may be given for balance.
Strategy:	Stand up: Move the trunk forward by flexion of the hips until the shoulders are above the right knee joint. Stand up while keeping weight on the leading leg. If possible: place the left foot next to the right (otherwise, do not move the foot, simply remain in lunge position)<br>Pause: Keep standing for 1 second.<br>Sit down: Return to half-knee position.<br>Repeat 8 times
Speed:	One stand-up per two to three seconds. One return per two to three seconds.
Correct trial:	Standing up through half-kneeling position while keeping the weight on the leading leg to the defined standing position which requires the child's trunk and lower extremities to be fully (maximally) extended, and slowly return on half knee
Incorrect trial:	Losing balance.<br>Standing up with an obviously asymmetrical posture during the test.<br>Unable to maintain a standing position for two seconds after standing up<br>swaying the trunk back and forth several times to initiate the task of standing up.<br>Sitting down abruptly without good control.
Adaptations<br>Initial starting position:	 If the child has contractures or wears a rigid orthosis, heel contact is not possible
