# Supplementary material for: Lower limb strength training in children with cerebral palsy – a randomized controlled trial protocol for functional strength training based on progressive resistance exercise principles
Source: BMC Pediatr. 2008 Oct 8;8:41. doi: 10.1186/1471-2431-8-41 (PMC2579291; doi:10.1186/1471-2431-8-41)
Supplement: Additional file 5 — Weekly training volumes and timing of the 8 RM test. This table describes the weekly training volumes and timing of the 8 RM test. [file 1471-2431-8-41-S5.pdf]

Leg Press: weekly training volumes and timing of the 8RM test

Week	Goal(s)	Set	Repetitions[a]	Load	Rest	8RM test
1-3	To introduce the children to circuit training	1	8	30-50% BW	120s	
	To explain the different exercises to the children	(2)[b]	8	30-50% BW	120s	
	To determine the initial starting position for each exercise	(3)	8	30-50% BW		
	To let the children practice the exercises with the correct technique					Week 3
4	To build up the training intensity	1	8	50% of 8RM[c]	120s	
		2	8	50% of 8RM	120s	
		3	8	75% of 8RM		
5	To build up the training intensity	1	8	50% of 8RM	120s	
		2	8	75% of 8RM	120s	
		3	8	100% of 8RM		
6-12	To initiate the strength training	1	8	50% of 8RM	120s	Week 6
		2	8	75% of 8RM	120s	Week 8
		3	8	100% of 8RM		Week 10

BW = body weight; RM = repetition maximum
a: Speed of repetition: extension movement: 2-3 seconds / flexion movement: 2-3 seconds
b: In the first 3 weeks, 1 to 3 sets may be performed depending on the time required to perform the exercise correctly.
c: An 8RM load corresponds to the amount of weight that can be moved on the leg-press through the available range of motion 8 times before needing a rest.

Loaded Sit-to-stand: weekly training volumes and timing of the 8RM test

Week	Goal	Set	Repetitions[a]	Load	Rest	8RM test
1-2	To introduce the children to circuit training	1	8	BW	120s	
	To explain the different exercises to the children	(2)[b]	8	BW	120s	
	To determine the initial starting position for each exercise	(3)	8	BW		
	To let the children practice the exercises with the correct technique					
3-4	To determine the initial starting position for each exercise	1	8	5-10% BW	120s	
	To let the children practice the exercises with the correct	(2)	8	5-10% BW	120s	
	technique	(3)	8	5-10% BW		Week 4
5	To slowly build up the training intensity	1	8	50% of 8RM[b]	120s	
		2	8	50% of 8RM	120s	
		3	8	50% of 8RM		
6	To slowly build up the training intensity	1	8	60% of 8RM	120s	
		2	8	60% of 8RM	120s	
		3	8	60% of 8RM		
7-12	To initiate the strength training	1	8	75% of 8RM	120s	*(Week 7)*[d]
		2	8	75% of 8RM	120s	Week 9
		3	8	75% of 8RM		*(Week 11)*[d]

BW = body weight; RM = repetition maximum
a: Speed of repetition: extension movement: 2-3 seconds / flexion movement: 2-3 seconds
b: In the first 4 weeks, 1 to 3 sets may be performed depending on the time required to perform the exercise correctly.
c: An 8RM load corresponds to the amount of weight that can be moved on the loaded sit-to-stand in the available range of motion 8 times before needing a rest.
d: The increase in training load for the loaded sit-to-stand in week 7 and 11 is estimated on the basis of the progress in 8RM on the leg-press between weeks 3 & 6 and 8 & 10 respectively. The minimal progress is set at 0% and the maximum at 10%.

Loaded game: weekly training volumes and timing of the 8RM test

Week	Goal	Set	Repetitions[a]	Load	Rest	8RM test
1-2	To introduce the children to circuit training	1	8	BW	120s	
	To clarify the different exercises to the children	(2)[b]	8	BW	120s	
	To determine the initial starting position for each exercise	(3)	8	BW		
	To let the children practice the exercises with the correct technique					
3-4	To determine the initial starting position on each exercise	1	8	2-5% BW	120s	
	To let the children practice the exercises with the correct	(2)	8	2-5% BW	120s	
	technique	(3)	8	2-5% BW		*Week 4* [d]
5	To build up the training intensity	1	8	15% of 8RM[c]	120s	
		2	8	15% of 8RM	120s	
		3	8	15% of 8RM		
6	To build up the training intensity	1	8	20% of 8RM	120s	
		2	8	20% of 8RM	120s	
		3	8	20% of 8RM		
7-12	To initiate the strength training	1	8	25% of 8RM	120s	*(Week 7)*[d,e]
		2	8	25% of 8RM	120s	Week 9 [d]
		3	8	25% of 8RM		*(Week 11)*[d]

BW = body weight; RM = repetition maximum
a: Speed of repetition: extension movement: 2-3 seconds / flexion movement: 2-3 seconds
b: In the first 4 weeks, 1 to 3 sets may be performed depending on the time required to perform the exercise correctly.
c: An 8RM load corresponds to the amount of weight that can be moved be on the loaded sit-to-stand through the available range of motion 8 times before needing a rest.
d: The increase in training load for the loaded game in weeks 4 and 9 is estimated on the basis of the 8RM test of the loaded sit-to-stand
e: The increase in training load for the loaded game in weeks 7 and 11 is estimated on the basis of the progress in 8RM on the leg-press between weeks 3 & 6 and 8 & 10 respectively. The minimal progress is set at 0% and the maximum at 10%.

Unloaded game: weekly training volumes

Week	Goal	Set	Repetitions[a]	Load	Rest
1-2	To introduce the children to circuit training	1	8	BW	120s
	To explain the different exercises to the children	(2)[b]	8	BW	120s
	To determine the initial starting position for each exercise	(3)	8	BW	
	To let the children practice the exercises with the correct technique				
3-12	To determine the initial starting position for each exercise	1	8	BW	120s
	To let the children practice the exercises with the correct technique	2	8	BW	120s
	To initiate the strength training	3	8	BW	

BW = body weight
a: Speed of repetition: extension movement: 2-3 seconds / flexion movement: 2-3 seconds
b: In the first 3 weeks, 1 to 3 sets may be performed depending on the time required to perform the exercise correctly.
